# The BcLAE1 is involved in the regulation of ABA biosynthesis in *Botrytis cinerea* TB-31

**DOI:** 10.3389/fmicb.2022.969499

**Published:** 2022-08-04

**Authors:** Zhao Wei, Dan Shu, Qun Sun, Dong-bo Chen, Zhe-min Li, Di Luo, Jie Yang, Hong Tan

**Affiliations:** ^1^CAS Key Laboratory of Environmental and Applied Microbiology, Environmental Microbiology Key Laboratory of Sichuan Province, Chengdu Institute of Biology, Chinese Academy of Sciences, Chengdu, China; ^2^Key Laboratory of Bio-Resources and Eco-Environment Ministry of the Education, College of Life Sciences, Sichuan University, Chengdu, China; ^3^University of the Chinese Academy of Sciences, Beijing, China

**Keywords:** *Botrytis cinerea*, abscisic acid, LAE1, secondary metabolism, gene regulation

## Abstract

Abscisic acid (ABA), as a classic plant hormone, is a key factor in balancing the metabolism of endogenous plant hormones, and plays an important role in regulating the activation of mammalian innate immune cells and glucose homeostasis. Currently, *Botrytis cinerea* has been used for fermentation to produce ABA. However, the mechanism of the regulation of ABA biosynthesis in *B. cinerea* is still not fully understood. The putative methyltransferase LaeA/LAE1 is a global regulator involved in the biosynthesis of a variety of secondary metabolites in filamentous fungi. In this study, we demonstrated that BcLAE1 plays an important role in the regulation of ABA biosynthesis in *B. cinerea* TB-31 by knockout experiment. The deletion of *Bclae1* caused a 95% reduction in ABA yields, accompanied by a decrease of the transcriptional level of the ABA synthesis gene cluster *Bcaba1-4*. Further RNA-seq analysis indicated that deletion of *Bclae1* also affected the expression level of key enzymes of BOA and BOT in secondary metabolism, and accompanied by clustering regulatory features. Meanwhile, we found that BcLAE1 is involved in epigenetic regulation as a methyltransferase, with enhanced H3K9me3 modification and attenuated H3K4me2 modification in Δ*Bclae1* mutant, and this may be a strategy for BcLAE1 to regulate ABA synthesis.

## Introduction

*Botrytis cinerea* is a notorious phytopathogenic ascomycete that causes gray mold disease in more than 200 host plant species and significantly damages large amounts of crops around the world every year (Dean et al., 2012; Fillinger and Elad, 2016). Although *B. cinerea* is a plant pathogen, this fungus produces several economically important compounds. The *B. cinerea* T4 and B05.10 genome sequences predicted 43 genes probably encoding key enzymes (KEs) for secondary metabolite (SM) biosynthesis, including 21 polyketide synthases (PKS), 1 chalcone synthase, 6 sesquiterpene cyclases (STC), 9 non-ribosomal peptide synthases (NRPS), 2 dimethylallyl tryptophan synthases (DMATS), and 5 diterpene cyclases (DTC; Amselem et al., 2011). It indicated that *B. cinerea* has the potential to produce them.

The classical phytohormone ABA, often referred to as the “stress hormone,” plays important roles in enhancing the tolerance abilities of plants to various kinds of abiotic or biotic stresses. The most common effect of ABA’s action is inhibition of growth and germination. It also regulates a number of processes such as seed ripening and dormancy, root growth, leaf aging, the transition from vegetative to generative growth, the activation of innate immune cells, and glucose homeostasis in mammals (Sakthivel et al., 2016). Thus, ABA has its broad application prospects in agriculture and medicine. Marumo et al. (1982) first reported that *B. cinerea* could synthesize ABA in 1982. Since then, other ABA-producing *B. cinerea* strains have also been confirmed and this plant pathogenic fungus has been studied as a model system for ABA production (Shi et al., 2017). Several *B. cinerea* strains have been particularly used for the fermentative overproduction of ABA at the industrial scale. In our laboratory, the *B. cinerea* wild-type strain TBC-6 (Tan et al., 1998) was isolated from wheat stems and leaves in southwest China. Strain improvements were performed on TBC-6, generating a series of *B. cinerea* mutants with different yields of ABA (Gong et al., 2014), such as TB-31, TB-3-H8, and TBC-A, which were used for biosynthesis regulation research and industrial production of ABA (Ding et al., 2016).

The putative methyltransferase LaeA/LAE1 has been demonstrated to be involved in regulating the biosynthesis of secondary metabolites (SMs) in most filamentous fungi (Bok and Keller, 2004). As a global regulator, studies have shown that LAE1 ortholog was characterized in *Penicillium chrysogenum*, *Fusarium fujikuroi*, and *Cochliobolus heterostrophus*, and their general function was found to be conserved in ascomycetes (Niehaus et al., 2018). LAE1 has the ability to activate biosynthesis potential, and its overexpression is considered to be an effective strategy to activate silent biosynthesis pathway and promote the discovery of new SMs in fungi. For example, a series of sorbicillinoids, including two new sorbitins, were found by overexpressing *lae1* in *Penicillium dipodomyis* (Yu et al., 2019). The overexpression of *lae1* upregulated the expression of chaetoglobosin biosynthetic gene cluster in *Chaetomium globosum*, which led to the discovery of a new cytochalasin (Jiang et al., 2016). In *B. cinerea* B05.10, LAE1 forms a trimeric protein complex with VEL1 and VEL2, which is very important in executing biological functions on morphogenesis under light control and the coordination of secondary metabolism. Studies have shown that BcVEL1 and BcLAE1 are required for the formation of oxalic acid (OA), tolerance to oxidative stress, and full virulence to infected plants. Meanwhile, BcVEL1 and BcLAE1 have strong effects on the expression of SM-related genes, including participation in phytotoxin biosynthesis. 16 SM key enzymes were differentially expressed in Δ*Bcvel1* and Δ*Bclae1* mutants compared with wild type (Schumacher et al., 2015).

LAE1 contains a hypothetical S-adenosine methyl-9-dependent (SAM) domain, which is similar to arginine methyltransferase. The truncation experiment showed that the N-terminal SAM-binding domain of LAE1 is necessary and has the function of nuclear localization (Bayram and Braus, 2012). Tudzynski et al. constructed mutants carrying a mutated *lae1* copy of the SAM domain in *Fusarium fujikura*, and found that the mutated gene copy *lae1*^SAM^ did not appear to be functional in contrast to wild type (Niehaus et al., 2018). Since no gibberellin acid (GA) gene expression and only very low product levels were detectable in the *lae1SAM-complemented* strain, indicating that the SAM domain is essential for the full activity of LAE1 in *F. fujikura*. The substrate for LAE1 methylation has not yet been determined, but there are more and more indications that LAE1 is involved in chromatin modification. For example, in *Aspergillus nidulans*, H4K12 acetylation of active gene clusters requires LAE1 to play a full role, most efficiently translating the acetylation signal into the noted increased transcriptional levels (Soukup et al., 2012). At the same time, in *Aspergillus luchuensis* mut. Kawachii, LaeA regulates citric acid production by regulating the expression of a putative citrate exporter-encoding gene *cexA via* changing methylation levels of the histones H3K4 and H3K9 (Kadooka et al., 2020). In addition, it has also been proved that LAE1 is resistant to the establishment of some gene cluster heterochromatin regions. Under the background of Δ*lae1*, the sterigmatocystin (ST) production level of wild type (WT) has been partially restored by knockout heterochromatin 1 (Hep1) or H3K9 methyltransferase ClrD (Reyes-Dominguez et al., 2010).

So far, the effect of LAE1 deletion or overexpression on ABA synthesis is unknown. In this work, we investigated the importance of the global regulator BcLAE1 on ABA biosynthesis in the high ABA-producing strain *B. cinerea* TB-31 and further presented its regulatory role in secondary metabolism and development. Meanwhile, we found that BcLAE1 is involved in the regulation of chromatin modification *B. cinerea* TB-31, which may be an important way of its function.

## Materials and methods

### Strains, plasmids, and ATMT

The ABA-hyperproducing *B. cinerea* mutant TB-31 was generated from multiple rounds of mutagenesis and screening initiated from TBC-6. Binary vectors pCBh1, pCBg1, and pCBsilent1 used in this study were constructed by our laboratory (Ding et al., 2015). *Escherichia coli* strain DH5ɑ was used as the host for transformation and genetic manipulation of plasmid DNA; 50 μg/ml kanamycin (Amresco,Solon, United States) was used to select positive colonies on LB agar plates. *B. cinerea* strains were grown on potato dextrose agar (PDA) slants at 25 C for 7 days. After conidia maturation, *Agrobacterium tumefaciens* EHA105-mediated transformation (ATMT) was performed, as described by Rolland et al. (2003), with some modifications. The recombinant plasmid was transfected into *A.tumefaciens* EHA105, 28 C, 220 rpm for 6 h to OD_600_ = 0.35–0.45. 100 μl conidia and 100 μl bacteria were mixed and spread on solid medium 3 with nitrocellulose membrane for 2 days. The membrane was then transferred to PDA solid medium for 3 days. 50 μg/ml hygromycin B (Sigma, St. Louis, United States) and 100 μg/ml glufosinate ammonium (Sigma) were used to select transformants on solid PDA plates. Further selection of gene overexpression transformants was performed by regenerating single conidia of the transformants on PDA plates supplemented with corresponding antibiotics.

### RNA and DNA extraction

The mycelium of the *B. cinerea* mutants and their control strain TB-31 cultured for 6 days were collected and washed with demineralized water, dried with filter paper, quenched in liquid nitrogen immediately, and ground into powder. Total RNA extraction was performed with E.Z.N.A. TM Fungal RNA Miniprep Kit (OMEGA, Cat # R6840-01) following the manufacturers’ instructions and on membrane DNaseI digestion was performed with E.Z.N.A. RNase-Free DNase I Set (OMEGA, Cat # E1091) for further DNA removal. The integrity and concentration of the extracted RNAs were quantified by NanoDrop spectrophotometer (Thermo Fisher Scientific, Waltham, MA, United States). Fungal genomic DNA was prepared according to E.Z.N.A.TM Fungal DNA Mini Kit (OMEGA, Cat # D3390-01).

### Construction of BcLAE1 transformants

A list of all primers used to prepare transformants and RT-qPCR is shown in Supplementary Table 1.

The knockout transformants were constructed using a double-combined PCR method to construct the *Bclae1* gene knockout fragment (Yu et al., 2004), and the upstream fragment (582 bp) and downstream fragment (672 bp) of *Bclae1* ORF were amplified with primer pairs *Lae1*-5-F1/−R1 and *Lae1-3*-F1/−R1, respectively. The hygromycin expression cassette fragment (*PoliC::hph*) was then amplified using the primer pair Hph-F1/Hph-R1. The knockout cassette was obtained by overlapping PCR with primer pair *Lae1*-5-F1/*Lae1*-3-R1, and then transformed into TB-31 protoplasts. The protoplasts were produced as previously described (Choquer et al., 2008). The construction of silencing transformants, complement transformants, and overexpression transformants was carried out by ATMT transformation method to transfer target vectors into corresponding strains. Transformants were selected on PDA containing hygromycin (50 μg/ml) or glyphosate (100 μg/ml) and purified by three rounds of subculture on PDA containing the same antibiotic selection.

### Extracellular ABA quantification

Single conidia of the *B. cinerea* transformants and the control strain were grown for 6–12 days on solid PDA 24-well plates at 25 C with 1.5 ml of PDA solid medium per well. The extracellular ABA that was secreted into the PDA medium was extracted with acetone, and the ABA contents of these extracted samples were measured with high-performance liquid chromatography (HPLC) using a commercial S-(+)-ABA (98% w/w, Lomon Bio Technology Co., Ltd., Sichuan, China) as the standard sample. The ABA standard curve was prepared by external standard method to detect samples. The Agilent 1,200 Pure Liquid Chromatography system (An Agilent 1,260 Infinity Quaternary Pump VL with An Agilent 1,260 Infinity Standard Autosampler and an Agilent 1,260 Infinity Variable Wavelength Detector) was used with a Luna® 5 μm C18(2) LC Column (Phenomenex, Cat # 00G-4,252-E0). The acetone-extracted samples were diluted to the same volume, and the ABA counts of these samples were determined based on their absorption at 254 nm (Supplementary Figure 1). All measurements were performed independently in triplicate.

### Quantitative RT-PCR

Quantitative reverse transcription-PCR (qRT-PCR) was performed to determine the relative expression levels of selected transcripts. The total RNA samples were utilized for gDNA-free cDNA synthesis with the ReverTra Ace-ɑ-® kit (Cat # FSK-101, TOYOBO, Japan). The synthesized cDNA was used as the template for PCR amplification of the selected genes with their corresponding primer pairs (Supplementary Table 1). The *B. cinerea* tubulin gene (BC1G_05600) was used to correct for sample-to-sample variation in the amount of RNA (Dulermo et al., 2010). Amplification was carried out by the CFX96 Real-Time PCR Detection System (BioRad, United States), using the TransStart Green qPCR SuperMix UDG (Transgen, China). The relative abundances of selected transcripts were calculated by the 2^−ΔΔCt^ method from the mean of three independent determinations of the threshold cycle (Schmittgen and Livak, 2008).

### Histone extraction and analysis

We modified the procedures for the extraction of histone described by Sidoli et al. (2016). Strains were grown on PDA plates at 25°C for 6 days. Mycelium was collected and ground into small tissues with Dounce homogenizer and resuspended in 50 ml of ice-cold NIB buffer (15 mm Tris-HCl [PH 8.5], 60 mm KCl, 15 mm NaCl, 5 mm MgCl2, 1 mm CaCl2, 250 mm sucrose, 1 mm PMSF, 1 ug/ml leupeptin, and 1 ug/ml pepstatin). The nuclear fraction was pellet by centrifugation (8,500 rpm), washed in the same buffer, and again recovered by centrifugation. Resuspend the nuclear fraction in ice-cold 0.4 M H2SO4 at a volume of 1:5. Shake at 4°C for 6 h, centrifuge the suspension (10,000 rpm) for 6 min, and transfer the supernatant to a new tube. Add cooled 100% TCA to the supernatant at a ratio of 1:3 (to obtain an optimal concentration of 33% TCA), invert the tube several times to mix, and incubate on ice overnight. After centrifugation again at 12,000 rpm for 30 min, precipitated histones were washed in ice-cold acetone twice and dried at room temperature. The purified histones were suspended in 100μl H2O and subjected to SDS-polyacrylamide gel electrophoresis and western blotting using antibodies against Histone H3 (catalog no.17168-1-AP; Proteintech), H3K4me (catalog no. 39498; Active Motif), H3K4me2 (catalog no.39141; Active Motif), H3K4me3 (catalog no.39060; Active Motif), H3K27me3 (catalog no. 39055; Active Motif), H3K14ac (catalog no. 39599; Active Motif), H3K9me (catalog no. 39887; Active Motif), H3K9me2 (catalog no. 39239; Active Motif), and H3K9me3 (catalog no. 39062; Active Motif).

### RNA-Seq

Undifferentiated hyphae of *B. cinerea* TB-31 and Δ*Bclae1* transformant were identified, and the hyphae were harvested after dark cultivation on potato dextrose agar (PDA) plates at 25°C for 6 days. Total RNA was extracted using E.Z.N.A.TM Fungal RNA Miniprep Kit according to the manufacturer’s protocol. RNA quality was assessed on an Agilent 2,100 Bioanalyzer (Agilent Technologies, Palo Alto, CA, United States) and checked using RNase-free agarose gel electrophoresis. After total RNA was extracted, eukaryotic mRNA was enriched by Oligo(dT) beads. After total RNA was extracted, prokaryotic mRNA was enriched by removing rRNA by Ribo-ZeroTM Magnetic Kit (Epicentre, Madison, WI, United States). Then, the enriched mRNA was fragmented into short fragments using fragmentation buffer and reversly transcribed into cDNA by using NEBNext Ultra RNA Library Prep Kit for Illumina (NEB #7530, New England Biolabs, Ipswich, MA, United States). The purified double-stranded cDNA fragments were end repaired, a base was added, and ligated to Illumina sequencing adapters. The ligation reaction was purified with the AMPure XP Beads (1.0X). Ligated fragments were subjected to size selection by agarose gel electrophoresis and poly merase chain reaction (PCR) amplified. The resulting cDNA library was sequenced using Illumina Novaseq 6,000 by Gene Denovo Biotechnology Co. (Guangzhou, China).

### Statistical analysis

Data were expressed as the mean ± SD. Histograms were generated using GraphPad Prism software, and *t*-tests for qPCR experiments were performed using Excel. Differences in ABA production of strains for different cultivation days were compared using SPSS v.16.0 (SPSS Inc., Chicago, IL, United States).

## Results

### Identification of the LAE1 ortholog in *Botrytis cinerea* TB-31

BcLAE1 was identified in the *B. cinerea* TB-31 genome database by SnapGene software analyses using the sequence of *B. cinerea* B05.10 LAE1 as query. The unique putative homolog of LAE1 showed a methyltransferase domain (100% identity). Like LAE1 in the *B. cinerea* B05.10, the open reading frame (ORF) of *Bclae1* in the *B. cinerea* TB-31 comprises 1,572 bp and encodes a protein of 327 aa (Schumacher et al., 2015). Meanwhile, the ORF is interrupted by six introns (181, 65, 73, 57, 64, and 148 bp). The only difference is that four bases “TACT” are deleted at the end of the 3′UTR (Supplementary Figure 2).

### BcLAE1 is required for ABA production in *Botrytis cinerea* TB-31

**Figure 1:**
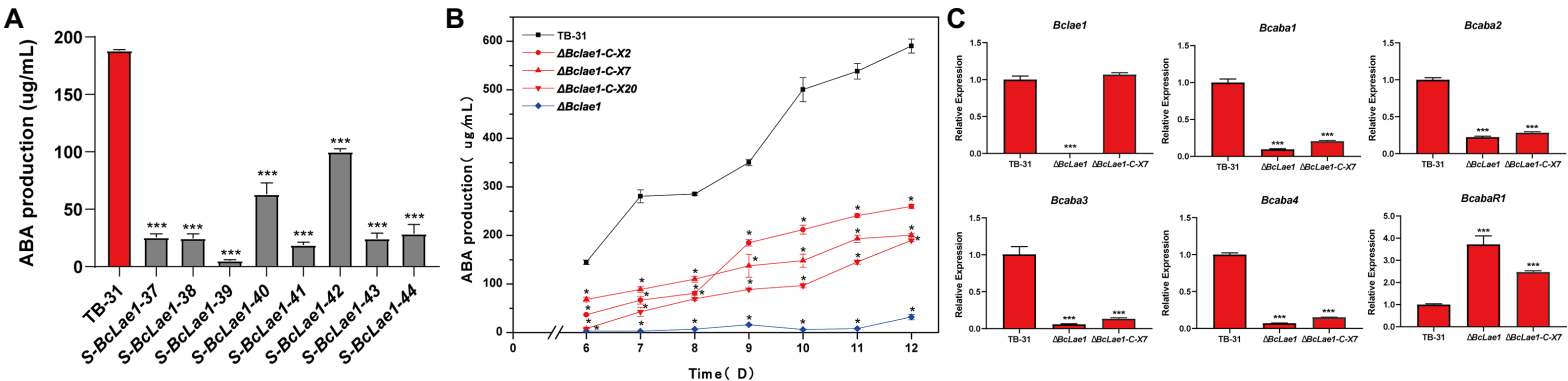
Deletion of *Bclae1* affects ABA synthesis. **(A)** Eight *Bclae1* gene silencing strains were randomly selected to grow on PDA. Samples for the quantitative determination of ABA production were collected at day 7. The error bars indicate the standard errors of the mean (SEM) for three replicate cultures (*n* = 3). Asterisks indicate significant differences in ABA production between selected silencing mutants and TB-31 (*p* < 0.001). **(B)** The Δ*Bclae1* mutant and three randomly selected Δ*Bclae1*-C mutants (Δ*Bclae1*-C-X2, Δ*Bclae1*-C-X7, Δ*Bclae1*-C-X20) were grown on PDA. Samples for the quantitative determination of ABA production were collected at 6–12 days. The error bars indicate the standard errors of the mean for three replicate cultures (*n* = 3). Asterisks indicate significant differences in ABA production between selected mutants and TB-31 (*p* < 0.05). **(C)** RT-qPCR examining the transcriptional levels of ABA gene cluster, *BcabaR1* and *Bclae1* in Δ*Bclae1* mutant, Δ*Bclae1*-C-X7, and TB-31. The relative transcriptional levels of selected genes were obtained after normalization to the constitutive tubulin reference gene (BC1G_05600) at 6 days. The relative values for selected genes transcription at 6 days in TB-31 were arbitrarily assigned as 100%. Shown are means and SEM, *n* = 3 independent biological replicates. ^***^p < 0.001 versus the same genes of the TB-31 group.

In order to study whether BcLAE1 will affect ABA synthesis, we previously constructed silencing strains of *Bclae1* gene based on the binary vector pCBSilent1 of gene silencing in the ATMT system. We randomly selected 8 *Bclae1* silencing transformants and cultured them for 7 days to determine ABA production. The results showed that the silencing of *Bclae1* gene reduced the production of ABA in different degrees (Figure 1A). To further identify the function of BcLAE1, we constructed the knockout transformant, the conserved domain was replaced by hygromycin expression cassette, and diagnostic PCR and qRT-PCR was used to confirm the deletion of *Bclae1* gene in Δ*Bclae1* transformant (Supplementary Figure 2). Then, the ABA yield of Δ*Bclae1* transformant and *B. cinerea* TB-31 was detected for 6–12 days, the results demonstrated that Δ*Bclae1* transformant hardly produced ABA, and its yield decreased by 95% compared with TB-31 at 12 days, indicating that BcLAE1 was essential for ABA synthesis (Figure 1B). After that, we focused on the expression patterns of the ABA biosynthesis gene cluster and positive regulator in *B. cinerea* strain: two hypothetical P450 monooxygenase coding genes (*Bcaba1* and *Bcaba2*; Takino et al., 2019), hypothetical FPP catalysis gene (*Bcaba3*; Shu et al., 2018), presumptive short-chain dehydrogenase/reducase coding genes (*Bcaba4*; Takino et al., 2019), and putative pathway-specific transcription factors (*BcabaR1*; Wang et al., 2018). The RNA of control strain TB-31 and Δ*Bclae1* transformant grown on PDA plates for 6 days were extracted and transformed into cDNA for RT-qPCR experiment. It was found that the expression level of *Bcaba1-4* gene in ABA biosynthesis gene cluster was significantly decreased except for pathway-specific transcription factors *BcabaR1* (Figure 1C).

**Figure 2:**
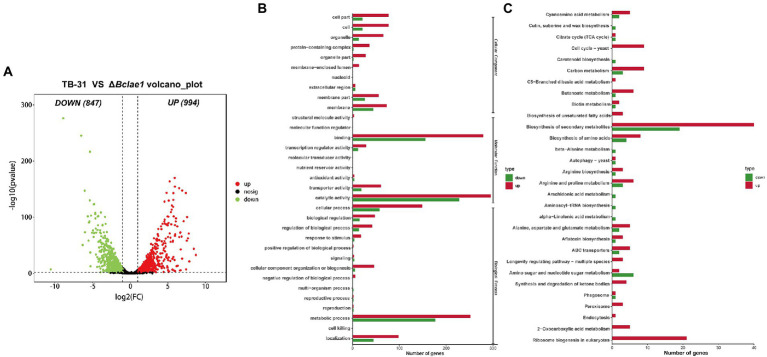
Genome-wide regulation of *Bclae1* deletion in *B. cinerea* TB-31. **(A)** The number of differentially expressed genes between TB-31 and Δ*Bclae1* mutant on day 6 of PDA culture. The criteria for the selection of DEGs were a log2 |fold change| ≥ 1, value of *p* < 0.05, and RPKM of at least one sample larger than 1. **(B,C)** Counts of differentially expressed genes significantly enriched to GO function classes and KEGG pathways, respectively.

To understand whether BcLAE1 supplementation can restore the ABA yield difference between Δ*Bclae1* and TB-31, the complement vector pCBg1-*Bclae1* was constructed and transferred into the Δ*Bclae1* transformant. Three BcLAE1 complement strains (Δ*Bclae1*-C) were randomly selected for 6–12 days of yield determination (Figure 2C). The results showed that the ABA yield was partially restored by *Bclae1* gene complementation, but it did not reach the level of the control strain TB-31. We selected complement transformant Δ*Bclae1*-C-X7 for RT-qPCR analysis. Although the expression level of *lae1* in Δ*Bclae1*-C-X7 was similar to that of control strain TB-31, the gene cluster *Bcaba1-4* was only slightly upregulated, and the expression level of *BcabaR1* was still in a high expression state. The expression pattern of *Bcaba1-4* was not significantly improved following the ectopic complementation of *Bclae1* gene (Figure 1C).

### Identification of differentially expressed genes between TB-31 and Δ*Bclae1*

In order to understand the impact of *Bclae1* deletion on the gene expression profiles, comparative transcriptome analysis was performed to identify the differentially expressed genes (DEGs) between the TB-31 and Δ*Bclae1* transformant by RNA-seq experiment. The undifferentiated mycelium spots were cultured on PDA solid medium for 6 days. Three biologically duplicated total RNA were extracted and marked respectively, and then, RNA-seq was performed. Based on the results of differential analysis, the differentially expressed genes (DEGs) were identified between the transformants using the threshold log2 |fold change| ≥ 1, value of *p* < 0.05, and RPKM value of at least one sample larger than 1. And there were a total of 1841 differentially expressed genes, of which 994 genes were upregulated and 847 genes were downregulated (Figure 2A). To determine the main biological functions of the DEGs, we performed GO and KEGG database analysis and annotation definition on the DEGs set to reveal the biological process affected by *Bclae1* deletion. The results demonstrated that compared with TB-31, the DEGs of Δ*Bclae1* transformant were significantly labeled as membrane-related in the cell components of GO database, which accounted for a larger proportion of the total differential genes. In molecular function and cell process, it is annotated as cofactor binding, oxidoreductase activity, catalytic activity, hydrolase activity, ribosome biogenesis, etc. (Figure 2B). In addition, the deletion of *Bclae1* had an obvious effect on the biosynthesis of secondary metabolites, ribosomal biogenesis, biosynthesis of amino acids, carbon metabolism, and other KEGG enrichment pathways (Figure 2C). We focus on significant transcriptional changes in metabolic networks related to carbon source transport and utilization and acetyl coenzyme A (acetyl-CoA) and ABA biosynthesis.

### The effect of *Bclae1* deletion on ABA synthesis pathway

The ABA synthesis pathway of *B. cinerea* is mainly divided into six stages, namely the transport and utilization of glucose, the synthesis of pyruvate, the synthesis of acetyl-CoA, the synthesis of isopentenyl pyrophosphate (IPP), the synthesis of farnesate pyrophosphate (FPP), and finally to ABA. As heterotrophs, glucose in cytoplasm is transported from outside the cells, and PDA medium contains a large amount of glucose, so we first analyzed the transcriptional level of sugar transporters and permeases. The results showed that the transcriptional levels of five putative sugar transporters and permeases increased significantly, namely BC1G_08389 (FC of 1.42), BC1G_11623 (FC of 3.55), BC1G_05673 (FC of 3.12), BC1G_12189 (FC of 3.01), and BC1G_05489 (FC of 1.79; Baruffini et al., 2006). Moreover, the transcriptional level of some β-glucosidases was also increased, such as BC1G_10877 (FC of 1.66), BC1G_11255 (FC of 2.1), BC1G_02551 (FC of 2.22), which indicated that the utilization efficiency of Δ*Bclae1* transformant to carbon source was enhanced compared with TB-31 (Supplementary Table 2.1).

The central metabolite acetyl-CoA is the link between the primary metabolic pathway and the secondary metabolic pathway, and is also the precursor of the synthesis of ABA in *B. cinerea*. The ultimate source of ABA is acetyl-CoA in the *B. cinerea* cytosol. Therefore, the genes involved in the biosynthesis and transportation of acetyl-CoA were analyzed. Pyruvate is an important node in the synthesis of glucose to acetyl-CoA. Glucose enters cells to generate pyruvate *via* glycolysis and pentose phosphate pathway (PPP). Except for the decreased transcriptional level of fructose-bisphosphate aldolase (FBA), the transcriptional levels of other rate-limiting enzymes in the glycolysis pathway did not change much. Meanwhile, there was little difference in the transcriptional levels of key genes in PPP, which indicated that the deletion of *Bclae1* has not greatly affected the carbon flux from glucose to pyruvate.

Cell cytosol pyruvate can be transformed into acetyl-CoA by two different pathways, the first being pyruvate dehydrogenase bypass (PDH bypass), it converts pyruvate to acetyl-CoA in cells (Shiba et al., 2007). And another pathway is that PDH catalyzes pyruvate decarboxylation and releases acetyl-CoA into mitochondrial matrix to participate in the tricarboxylic acid (TCA) cycle (Guest et al., 1989). In contrast, there was no significant difference in the transcriptional levels of genes encoding different components of PDH between TB-31 and Δ*Bclae1* transformant, but the transcriptional level of acetyl-CoA synthetase was elevated, which is conducive to increasing the content of acetyl-CoA in the cytosol. In most fungi, acetyl-CoA can also be produced by β-oxidation of fatty acids in mitochondria and peroxisomes. The transcriptional level of the 21 genes involved in the β-oxidation of mitochondria and peroxisomes had little change except that the Enoyl-CoA hydratase increased slightly, so the content of acetyl-CoA was no significant change by the β-oxidation of fatty acids. Since the TCA cycle is the main metabolic pathway to mitochondrial consumption of acetyl-CoA, we also analyzed the key genes in the TCA cycle. The data showed that the transcriptional level of citrate synthase increased, but pyruvate was the main donor of oxaloacetate in mitochondria, and the decreased transcriptional level of pyruvate carboxylase reduced the ability of pyruvate to produce oxaloacetate, which may affect the synthesis of citrate. At the same time, in the glyoxylic acid cycle (GYC), the transcriptional level of isocitrate lyase decreased, thus reducing the ability of isocitrate to synthesize glyoxylic acid and succinate, which may interfere with glyoxylate consumption of acetyl-CoA to synthesize apple acid. These changes may impede the fluidity of the TCA cycle, resulting in impaired acetyl-CoA utilization in the TCA cycle. In addition, acetyl-CoA in the cytoplasm is also the precursor of cellular lipids (such as fatty acids; Camões et al., 2015). Compared with TB-31, the transcriptional levels of genes encoding fatty acid synthesis-related enzymes, such as fungi-type fatty acid synthase and [acyl-carrier-protein] S-malonyltransferase, were not significantly altered in Δ*Bclae1* transformant. Carnitine/acetylcarnitine shuttle system is very important for the intracellular transport of acetyl-CoA between mitochondria, peroxisomes, and cytoplasm (Strijbis et al., 2010; Lee et al., 2011). We analyzed three possible carnitine O-acetyltransferases and found that the transcriptional level also did not change significantly (Supplementary Table 2.2). In conclusion, the accumulation of cytosol acetyl-CoA was not greatly affected by the deletion of the *Bclae1* gene.

**Figure 3:**
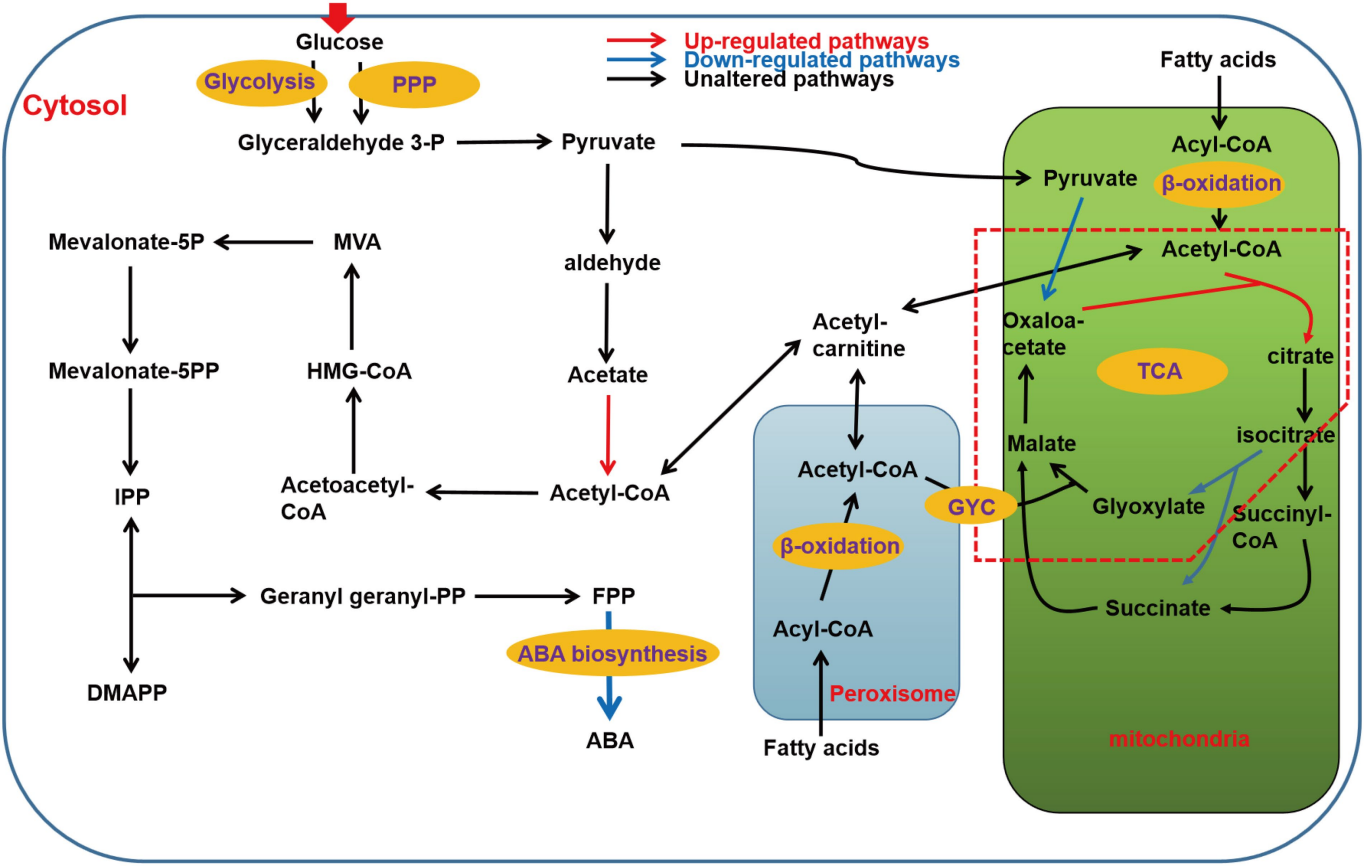
Schematic of metabolic pathways associated with ABA production affected by *Bclae1* deletion. The red arrows indicate upregulated pathways, blue arrows indicate downregulated pathways, and black arrows indicate pathways that were not observably regulated. The red dashed box annotates the GYC pathway (log2 |fold change| ≥ 1, value of *p* < 0.05 and RPKM of at least one sample larger than 1).

In *B. cinerea*, acetyl-CoA can synthesize IPP and dimethylallyl-diphosphate (DMAPP) through the MVA pathway (Oritani and Kiyota, 2003), which are precursors for terpenoid backbone biosynthesis. This is followed by the biosynthesis of FPP, which is a direct precursor for the biosynthesis of ABA from IPP (Dewick, 2002). RNA-Seq analysis showed that there were no significant changes in the transcriptional level of all enzymes from acetyl-CoA to FPP. However, the transcriptional level of ABA synthetic gene cluster changed greatly, in which the FPKM average of BC1G_07529 (*Bcaba4*) gene decreased from 139 to 20, the FPKM average of BC1G_07530 (*Bcaba2*) gene decreased from 666 to 278, the FPKM average of BC1G_07531 (*Bcaba1*) gene decreased from 1743 to 290, and the FPKM average of BC1G_07534 (*Bcaba3*) gene decreased from 504 to 53, which indicated that the deletion of *Bclae1* was mainly aimed at the decrease of transcriptional level of ABA synthetic gene cluster (Supplementary Table 2.3). Since Bcaba1-4 plays an important role in the final synthesis of FPP to ABA, combined with the above RT-qPCR data, we believe that this may be the key factor of decreased ABA production in the Δ*Bclae1* transformant (Figure 3).

### Transcriptional level of KEs in secondary metabolism is affected

**Figure 4:**
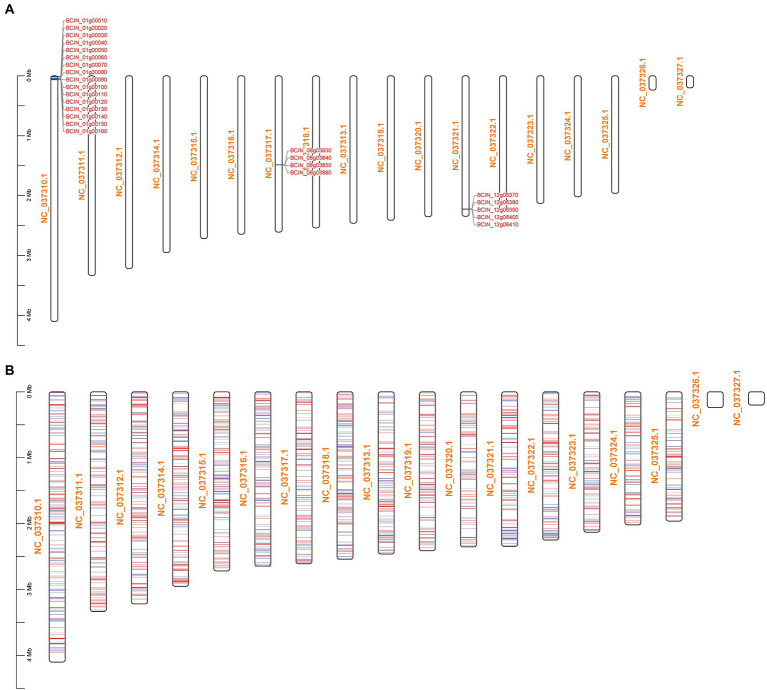
Schematic of DEGs mapping across chromosomes. NC_037310.1-NC_037327.1 are the numbers of the 16 chromosomes of *B. cinerea*. **(A)** BCIN_01g00010-BCIN_01g00160 are BOA synthetic gene cluster, BCIN_08g03830-BCIN_08g03880 are ABA synthetic gene cluster, and BCIN_12g06370-BCIN_12g06410 are BOT synthetic gene cluster. **(B)** The mapping of all DEGs on chromosomes, the red line represents genes with upregulated transcription level, and the blue line represents genes with downregulated transcription level.

The KEGG pathway enrichment results showed that BcLAE1 has a significant regulatory effect on secondary metabolism; so in addition to ABA synthesis, we also analyzed differentially expressed genes for 43 key enzymes of secondary metabolism in transcriptome data (Supplementary Table 2.4). KEs with FPKM less than 1 were eliminated, and the transcriptional level of 6 KEs was obviously changed (value of *p* < 0.05). The expression level of the sesquiterpene synthase gene *BcSTC3* was upregulated (FC of 1.1). Although the corresponding compound has not been discovered, a new sesquiterpene structure associated with eremophil-9-ene was found in the presence of chemical induction of copper sulfate with the increased expression of *BcSTC3* and *BcSTC4* gene (Pinedo et al., 2016). The expression level of another sesquiterpene cyclase *BcSTC1/Bcbot2* gene was significantly decreased (FC of-4.6). *Bcbot2* is a key gene for the synthesis of sesquiterpenoid botrydial (BOT), which induces hypersensitivity and uses host defense mechanisms to generate necrotic cells (Simon et al., 2013). We also analyzed the BOT synthetic gene cluster and found that not only the *Bcbot2* gene but also the other four genes (*Bcbot1-5*) were significantly downregulated. At the same time, the expression level of *BcPKS13*, a putative synthesis gene of polyketide derivative 1,8-Dihydroxynaphthalene (DHN)-melanin (Schumacher, 2016), was also downregulated (FC of-1.2). The transcriptional levels of the other two differential polyketide synthase (PKS) genes, *BcPKS19* (FC of-1.2) and *BcPKS8* (FC of-2), were downregulated like the non-ribosomal peptide synthase gene *BcNRPS5* (FC of-1.2), but their corresponding compounds have yet to be identified. Meanwhile, we also found that the transcriptional levels of most botcinic acid (BOA; Porquier et al., 2019) synthesis genes were significantly downregulated (value of *p* < 0.05), including *Bcboa3-5* genes in BOA gene cluster A and *Bcboa10, 12, 16, 17* genes in BOA gene cluster B. Moreover, the FPKM of key genes *BcPKS6* and *BcPKS9* and other genes in the cluster also decreased, suggesting that BcLAE1 is involved in the regulation of the transcriptional level of the whole cluster. It was obviously that BcLAE1 affects the expression of SM-related gene expression in *B. cinerea* TB-31, and shows a preference for the whole cluster regulation. After that, we mapped the ABA gene cluster, the BOT gene cluster, the BOA gene cluster, and all the differential genes to the chromosomes of *B. cinerea,* respectively (Figure 4). It was found that some of the differential expression genes caused by *Bclae1* deletion showed aggregation.

To confirm the expression pattern of Δ*Bclae1* transformant genes in the RNA-Seq, we selected *Bcbot1*, *Bcbot2*, *Bcbot3*, *Bcboa3*, *BcPKS13*, *Bccit3*, *Bcfas1*, *Bcfas2*, *BcACS*, *BcFBA*, *BcICL*, *BcMLS*, *BcNRPS5*, *BcPKS19*, *BcPYC*, BC1G_05489, and BC1G_11623 genes to design primers, and used the isolated RNA for RT-qPCR verification (Supplementary Figure 3). The results indicated that the selected genes basically conformed to the expression pattern predicted by the RNA-Seq.

### Overexpression of *Bclae1* did not increase ABA production in *Botrytis cinerea* TB-31

**Figure 5:**
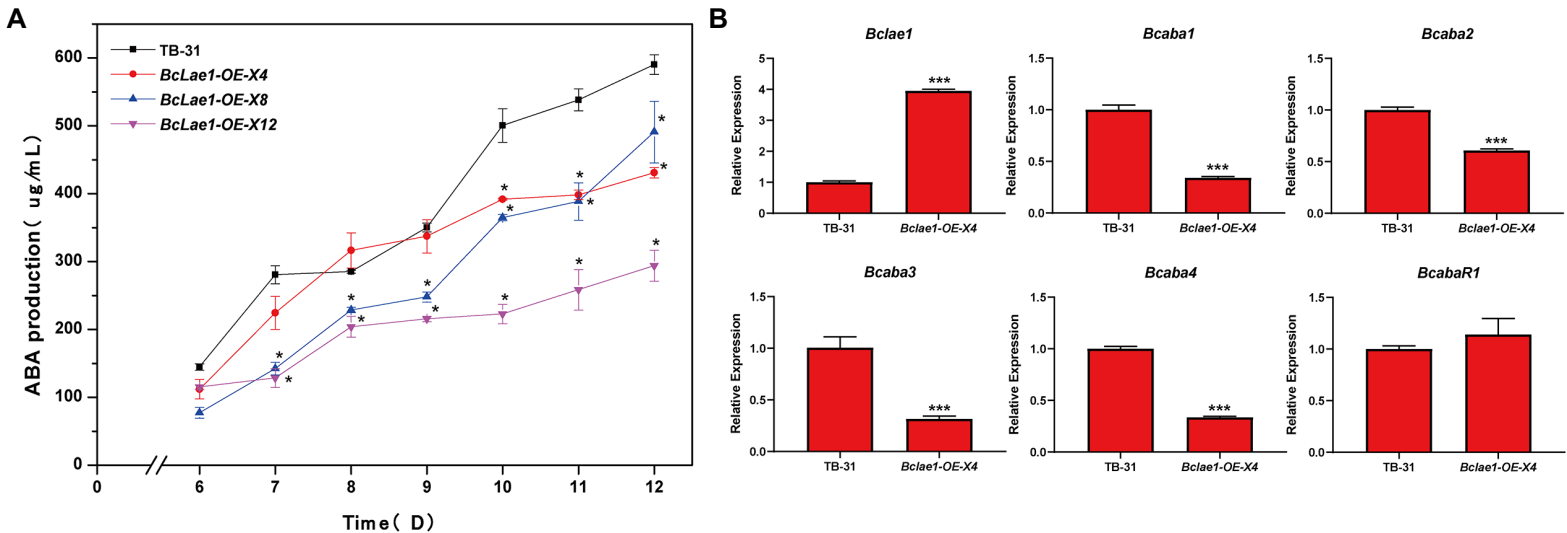
Overexpression of *Bclae1* reduced ABA synthesis in *B. cinerea* TB-31. **(A)** The TB-31 and 3 randomly selected *Bclae1*-OE mutants (*Bclae1*-OE-X4, *Bclae1*-OE-X8, *Bclae1*-OE-X12) were grown on PDA. Samples for the quantitative determination of ABA production were collected at 6–12 days. The error bars indicate the standard errors of the mean for three replicate cultures (*n* = 3). Asterisks indicate significant differences in ABA production between selected mutants and TB-31 (*p* < 0.05). **(B)** RT-qPCR examining the transcriptional levels of ABA gene cluster, *BcabaR1* and *Bclae1* in *Bclae1*-OE-X4 and TB-31. The relative transcriptional levels of selected genes were obtained after normalization to the constitutive tubulin reference gene (BC1G_05600) at 6 days. The relative values for selected genes transcription at 6 days in TB-31 were arbitrarily assigned as 100%. Shown are means and SEM, *n* = 3 independent biological replicates. ^***^p < 0.001 versus the same genes of the TB-31 group.

Based on the data of silence strains and knockout strain, it was found that the deletion of *Bclae1* gene reduced the yield of ABA. Considering that BcLAE1 has a significant effect on the synthesis of secondary metabolites, we further analyzed the effect of constitutive expression of *Bclae1* on ABA production in *B. cinerea* TB-31. We constructed the overexpression transformant *Bclae1*-OE by using the overexpression vector pCBh1 with hygromycin resistance gene. The obtained 12 overexpression transformants were cultured for 7 days for ABA production detection, and it was found that the ABA production of all transformants decreased compared with TB-31 transferred into pCBh1 empty vector (Supplementary Figure 4). Considering whether the ABA yield would recover at the later stage of strain culture, three *Bclae1*-OE transformants were randomly selected for 6–12 days ABA yield determination. The results showed that even if the three *Bclae1*-OE transformants were cultured for 12 days, ABA production was still reduced (Figure 5A). Transcriptional levels of ABA synthesis gene cluster and pathway-specific transcription factor were detected in *Bclae1*-OE-X4 transformant. The results showed that the expression of the *Bclae1* gene was significantly increased compared with the control strain TB-31, while the expression of *Bcaba1-4* gene was decreased, and there was no significant difference in the expression of *BcabaR1* (Figure 5B). This indicates that overexpression of *Bclae1* is not beneficial for ABA synthesis in *B. cinerea* TB-31.

### The SAM domain of BcLAE1 is involved in ABA biosynthesis in *Botrytis cinerea* TB-31

**Figure 6:**
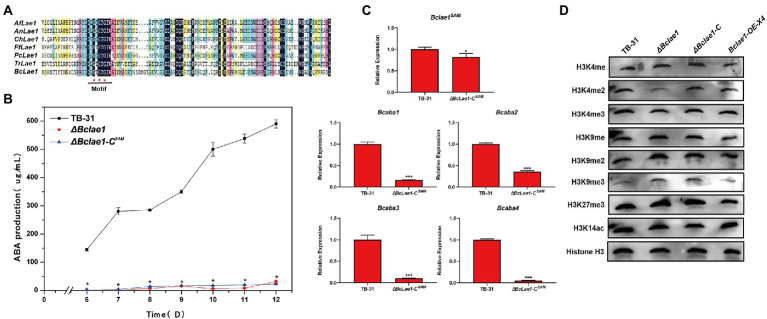
The SAM domain of BcLAE1 is essential for its full activity. **(A)** Multiple sequence alignment of the LaeA/ LAE1 SAM domain from *Aspergillus fumigatus* LaeA, *Aspergillus nidulans* LaeA, *Cochliobolus heterostrophus* LAE1, *Fusarium fujikuroi* LAE1, *Cochliobolus heterostrophus* LAE1, *Trichoderma reesei* LAE1, and *B. cinerea* LAE1. Black, pink, blue, and yellow represent 100%, greater than 75%, greater than 50%, and greater than 30% homology levels, respectively. The red ^*^ marked the glycine that was replaced for alanine. **(B)** The TB-31, Δ*Bclae1* mutant, and Δ*Bclae1*-C^SAM^ mutant were grown on PDA. Samples for the quantitative determination of ABA production were collected at 6–12 days. The error bars indicate the standard errors of the mean for three replicate cultures (*n* = 3). Asterisks indicate significant differences in ABA production between selected mutants and TB-31 (*p* < 0.05). **(C)** RT-qPCR examining the transcriptional levels of ABA gene cluster and *Bclae1* in Δ*Bclae1*-C^SAM^ and TB-31. The relative transcriptional levels of selected genes were obtained after normalization to the constitutive tubulin reference gene (BC1G_05600) at 6 days. The relative values for selected genes transcription at 6 days in TB-31 were arbitrarily assigned as 100%. Shown are means and SEM, *n* = 3 independent biological replicates. ^*^p < 0.05/^***^p < 0.001 versus the same genes of the TB-31 group. **(D)** Western blots for H3K4me1/2/3, H3K9me1/2/3, H3K27me3, and H3K14ac global status in the histone extracts isolated from the 6 days cultures of the TB-31, Δ*Bclae1*, Δ*Bclae1*-C (X7), and *Bclae1*-OE-X4 mutants. Anti-H3 was used as a loading control.

Since LAE1 can also act as a putative methyltransferase to affect chromatin modification changes to regulate secondary metabolic synthesis, to illuminate whether BcLAE1 plays a role as a methyltransferase in ABA synthesis, we studied the conserved sites of the BcLAE1 SAM domain. We selected six functionally characterized LaeA/ LAE1 orthologs from ascomycetes for comparison with BcLAE1, namely *Aspergillus fumigatus* LaeA (Dagenais et al., 2010), *Aspergillus nidulans* LaeA (Khan et al., 2020), *Cochliobolus heterostrophus* LAE1 (Wu et al., 2012), *Fusarium fujikuroi* LAE1 (Wiemann et al., 2010), *Penicillium chrysogenum* LaeA (Kosalková et al., 2009), and *Trichoderma reesei* LAE1 (Karimi-Aghcheh et al., 2013; Figure 6A). The methyltransferase domain was demonstrated and three conserved glycine residues G^90^, G^92^, and G^94^ were marked. Then, the three glycine were replaced by alanine to construct the complement vector pCBg1-*Bclae1*^G90,92,94A^. The vector was transferred into Δ*Bclae1* transformant and the ABA yield of the Δ*Bclae1*-C^SAM^ transformant was measured from 6 to 12 days; the results showed that Bc*lae1*^G90,92,94A^ gene did not restore the yield of ABA, and the production data were basically consistent with the Δ*Bclae1* transformant (Figure 6B). We further analyzed the expression of Bc*lae1*^G90,92,94A^ in Δ*Bclae1*-C^SAM^ transformants by RT-qPCR validation, and found that the expression levels of *Bclae1*^G90,92,94A^ in the transformant was close to that of *Bclae1* in TB-31. At the same time, we examined the expression of ABA synthesis gene clusters and pathway-specific transcription factors, and the results were similar to the Δ*Bclae1* transformant (Figure 6C). These data suggested that the SAM domain of BcLAE1 plays important roles in ABA biosynthesis in *B. cinerea* TB-31.

### The deletion of *Bclae1* affects H3K9me3 and H3K4me2 modification

The above experiments suggest that BcLAE1 may play the role of methyltransferase. Although the substrate of LAE1 methylation is not clear, more and more indirect evidence suggests that LAE1 may affect expression of SMs gene clusters through the apparent genetic modification of chromatin structure. For example, HstD/AoHst4 may be involved in epigenetic regulation caused by laeA gene expression to coordinate fungal development and secondary metabolism (Kawauchi et al., 2013).

In order to study the possible effect of BcLAE1 on histone modification, we extracted histone of TB-31, Δ*Bclae1*, Δ*Bclae1*-C, and *Bclae1*-OE-X4 transformants; then, the modification levels of H3K4me1/2/3, H3K9me1/2/3, H3K27me3, and H3K14ac were analyzed by Western blot (WB). The results showed that there was no significant change in *Bclae1*-OE-X4 transformant compared with TB-31. The modification levels of H3K4me, H3K4me3, H3K9me2, H3K9me2, H3K27me3, and H3K14ac in Δ*Bclae1* were also not significantly affected, but the level of H3K4me2 was decreased and the level of H3K9me3 was increased, suggesting that BcLAE1 was involved in chromatin modification regulation (Figure 6D). The Δ*Bclae1*-C transformant partially recovered the level of H3K4me2, but did not completely reduce the accumulation of H3K9me3. As heterochromatin is important for maintaining genome integrity, it is characterized by histone H3K9 methylation and tail hypoacetylation (Steinhauf et al., 2014). In other words, the loss of *Bclae1* may lead to changes in the heterochromatin landscape in the genomic region that regulate fungal development and secondary metabolism.

### Deleting *Bclae1* changes the strain development and differentiation program

**Figure 7:**
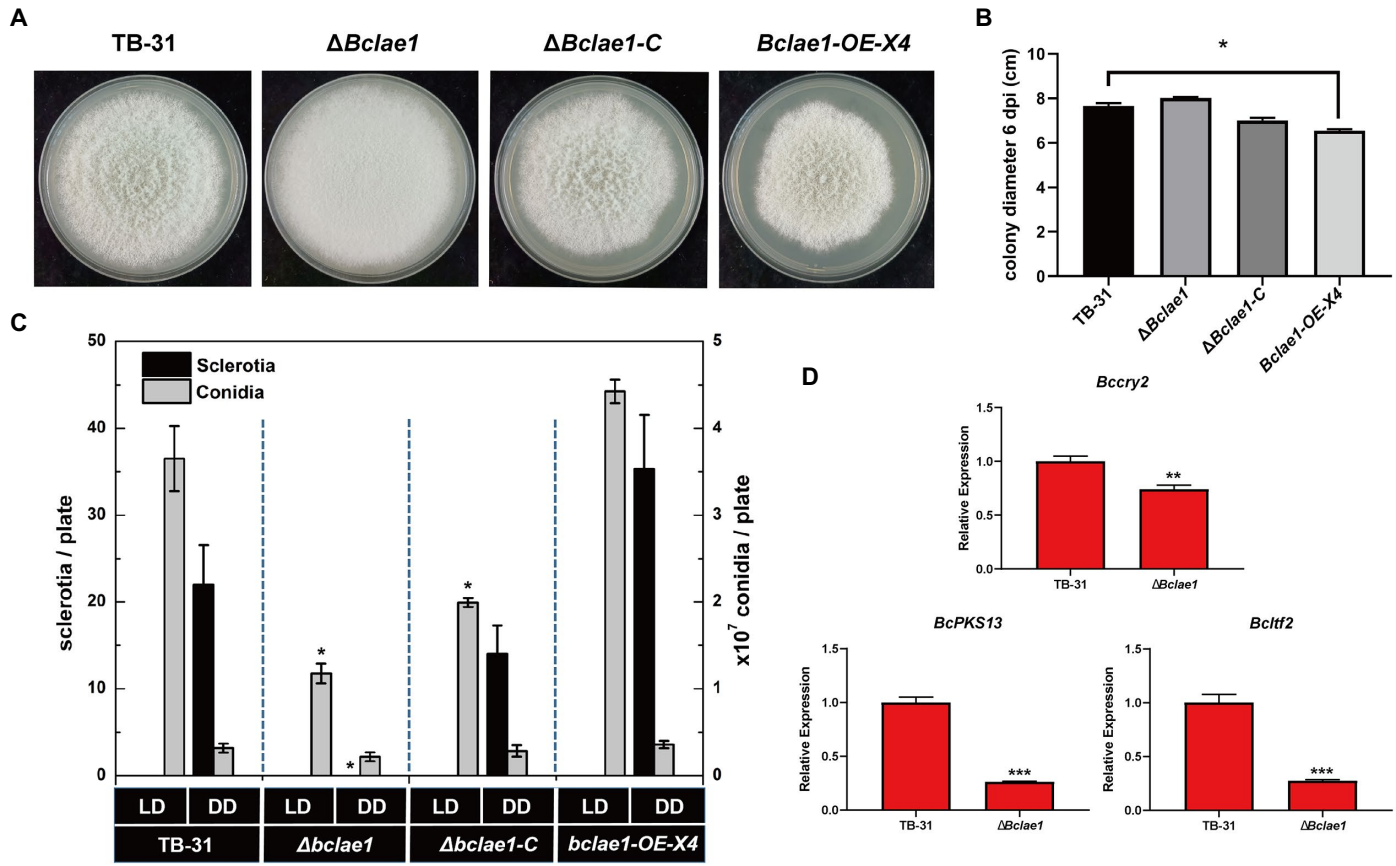
Deleting *Bclae1* changes the growth, morphology, and reproductive development. **(A)** The TB-31, Δ*Bclae1*, Δ*Bclae1*-C (X7), and *Bclae1*-OE-X4 mutants were photographed after 6 days of PDA growth under DD culture conditions, and the colony diameters were calculated **(B)**. Shown are means and SEM, *n* = 3 independent biological replicates. ^*^p < 0.05 versus the colony diameter of the TB-31 group. **(C)** Under DD or LD conditions, TB-31, Δ*Bclae1*, Δ*Bclae1*-C (X7), and *Bclae1*-OE-X4 mutants were counted for sclerotia and conidia numbers after 14 days of PDA growth. Shown are means and SEM, *n* = 3 independent biological replicates. ^*^p < 0.05 versus the TB-31 group. **(D)** RT-qPCR examining the transcriptional levels of *Bccry2*, *BcPKS13*, and *Bcltf2* in TB-31 and Δ*Bclae1*. Shown are means and SEM, *n* = 3 independent biological replicates. ^**^p < 0.01/^***^p < 0.001 versus the same genes of the TB-31 group.

LAE1 is involved in the growth, morphology and reproductive development of fungi (Bayram and Braus, 2012), the TB-31, Δ*Bclae1*, Δ*Bclae1*-C, and *Bclae1*-OE-X4 transformants were cultivated to track the morphology of hyphal, colony growth. When cultured to the 6th day, the Δ*Bclae1* transformant showed excessive aerial hyphal formation, which gave the colony as “marshmallow” appearance. At the same time, the hyphal color of the Δ*Bclae1* transformant did not show gray, but turned white. The Δ*Bclae1*-C transformant made their mycelium morphology and color close to TB-31, which indicated that BcLAE1 was indeed involved in the regulation of mycelial development. Furthermore, the colony diameter of *Bclae1*-OE transformant became smaller than that of TB-31 (Figure 7A). Although the morphology of hyphae did not change, the growth rate was slowed down (Figure 7B). The transcriptome data (Supplementary Table 2.5) revealed that the gene transcriptional levels of the putative UV-light-sensing cryptochrome *Bccry2* (Cohrs and Schumacher, 2017) were upregulated in Δ*Bclae1* compared to TB-31. The gene transcriptional levels of the key melanin synthesis enzyme *BcPKS13* and the conidial regulator *Bcltf2* (Cohrs et al., 2016) were downregulated in Δ*Bclae1* (value of *p* < 0.05). RT-qPCR analysis indicated that the expression levels of *BcPKS13* and *Bcltf2* were consistent with the RNA-Seq except for *Bccry2* (Figure 7D).

We also detected the growth of conidia and sclerotia under different light conditions. Conidia, as the source of inoculation for host infection, are mainly produced under light conditions. As the female parent of sexual reproduction, the sclerotia is mainly produced in the absence of light. Since it takes longer for sclerotia to form than conidia, the number of conidia and sclerotia of transformants was detected after 2 weeks of culture. Under continuous darkness (DD) and 12-h light and 12-h dark (LD) photoperiod, the Δ*Bclae1* transformant did not form sclerotia under DD photoperiod, but only a small number of conidia formed (Figure 7C). However, the *Bclae1*-OE-X4 transformant formed more sclerotinia and conidia than control strain TB-31. Due to the lack of sclerotia formation, the Δ*Bclae1* transformant was female sterile. At the same time, under the condition of LD photoperiod, the number of conidia decreased a lot compared with the control strain TB-31. The Δ*Bclae1*-C transformant made up for the lack of sclerotia formation function and increased the conidia production compared with the Δ*Bclae1* transformant. Overall, LAE1 is critical to the differentiation program under light control.

## Discussion

*Botrytis cinerea*, as a filamentous ascomycete, is the pathogen that causes gray mold infection in more than 1,000 plants (Dean et al., 2012). Therefore, most of the studies on *B. cinerea* are mainly related to virulence factors. For example, BcReg1 in *B. cinerea* is involved in regulating the production of plant toxins (Michielse et al., 2011). Reboledo et al. (2020) studied genome-wide transcriptional profiling of *B. cinerea* during different infection stages. In addition, the two secondary metabolites BOT and BOA produced by *B. cinerea* are also the focus of research (Dalmais et al., 2011; Malmierca et al., 2016). In addition to phytotoxins, *B. cinerea* can also secrete a variety of secondary metabolites, one of the most famous secondary metabolites is ABA. LAE1/LaeA is a secondary metabolic regulator, and its deletion or overexpression affects the expression patterns of multiple SM genes. Studies have shown that LaeA regulates penicillin production in *P. chrysogenum* (Kosalková et al., 2009). In addition, overexpression of *laeA* orthologs also resulted in higher production of trichothecenes in *F. Graminearum* (Kim et al., 2013), aflatoxin in *Aspergillus flavus* (Kale et al., 2008), T-toxin in *C. heterostrophus* (Wu et al., 2012), and pigments in *Monascus pilosus* (Lee et al., 2013). Up to now, there has been no report on the effect of LAE1 on the synthesis of ABA in *B. cinerea*. In this study, *B. cinerea* TB-31 with high ABA yield was used to observe the effect of *Bclae1* deletion on ABA production, and the differential genes in ABA synthesis pathway were screened by RNA-seq data. It was found that BcLAE1 significantly affected the expression of ABA synthesis gene cluster. At the same time, BcLAE1 plays its own methyltransferase function and participates in the regulation of histone modification, which may also be a way for BcLAE1 to regulate ABA synthesis.

In order to study the effect of BcLAE1 on ABA synthesis, we first constructed *Bclae1* silencing strain. Yield assays confirmed that *Bclae1* silencing resulted in decreased ABA synthesis. Then, we constructed the *Bclae1* knockout mutant, after 6–12 days of cultivation, it was found that the deletion of *lae1* seriously disrupted the ABA synthesis ability, accompanied by a decrease of the transcriptional level of the ABA synthesis gene cluster *Bcaba1-4*. In a previous study, we found a novel Cys2His2 transcription factor (TF), BcabaR1, which is a positive regulator of ABA biosynthesis in *B. cinerea*. BcabaR1 regulates the transcriptional levels of ABA synthase genes by binding specifically to the promoter region of *Bcaba1-4* genes (Wang et al., 2018). In this study, the transcriptional level of *BcabaR1* gene in Δ*Bclae1* was upregulated compared with TB-31, which did not drive the transcriptional level of the entire ABA gene cluster to increase, indicating that there are other unknown regulatory ways of ABA synthesis.

In some fungi such as *Fusarium fujikuroi*, although the deletion of *lae1* gene led to a sharp decrease in the expression of SM genes, the overexpression of *lae1* gene also led to the increase of some SM genes expression, which led to the improvement of product level (Niehaus et al., 2018). Therefore, the overexpression of *laeA/ lae1* has also been successfully used to activate some gene clusters. LAE1, as the main focus of fungal strain improvement, we also overexpressed *Bclae1* in TB-31. However, the yield of ABA decreased after overexpression of *Bclae1*. The transcriptional level of the ABA gene cluster was downregulated in *Bclae1*-OE-X4 transformant, indicating that the overexpression of *Bclae1* is not beneficial to the biosynthesis of ABA in *B. cinerea* TB-31. We speculate that the reason for this phenomenon is that the regulation triggered by LAE1 is global, which is affected by many unknown factors, such as growth and development. LAE1 does not regulate specific secondary metabolism, and the yield of a single secondary metabolism is not positively correlated with the expression level of *lae1*. Although overexpression of *lae1* resulted in the overproduction of some secondary metabolites, it did not lead to increased expression levels of all KEs in secondary metabolism (Kim et al., 2013; Niehaus et al., 2018). At the same time, the product changes caused by lae1 overexpression are different in different strains, so how LAE1 increases the production of a specific secondary metabolite needs to be studied.

To further understand how the effect of *Bclae1* deletion on ABA synthesis, we compared RNA-seq data to analyze the differentially expressed genes in ABA synthesis pathway. The Δ*Bclae1* transformant has significantly improved the transport and utilization efficiency of glucose, which is definitely favorable for the synthesis of acetyl-CoA. However, the transcriptional levels of genes involved in pyruvate synthesis, acetyl-CoA synthesis, IPP synthesis, and FPP synthesis did not change significantly, and only the expression level of *Bcaba1-4* in the ABA synthesis gene cluster was severely downregulated. Therefore, we believed that this was the direct cause of the decreased ABA synthesis in the Δ*Bclae1* transformant.

In addition to the secondary metabolite ABA, we also paid attention to the expression of the other 43 key enzymes in the secondary metabolism. The transcriptional levels of six key enzyme genes were changed, which were *BcSTC1/Bcbot2* (FC of-4.6), *BcSTC3* (FC of 1.1), *BcPKS13* (FC of-1.2), *BcPKS19* (FC of-1.2), *BcPKS8* (FC of-2), and *BcNRPS5* (FC of-1.2). The corresponding compounds of the BcSTC3, BcPKS19, BcPKS8, and BcNRPS5 have yet to be identified. *Bcbot2* is the key gene for the synthesis of the virulence factor botrydial (BOT). We found that the entire BOT synthesis gene cluster, including *Bcbot2*, was downregulated at the transcriptional level like the ABA synthesis gene cluster. This downregulation even affected *Bcbot6* and *Bcbot7* (Porquier et al., 2016), which are 10 kb away from the BOT gene cluster. Bcbot6 is a positive regulatory transcription factor of the BOT gene cluster, and Bcbot7 is predicted to be a dehydrogenase that may be involved in the transformation of BOT to dihydrobotrydial. Studies have shown that *Bcbot1-Bcbot5* genes and the newly discovered *Bcbot7* gene are all dependent on the regulation of BcBot6, but the deletion of *Bclae1* down-regulates all *Bcbot1-7* genes, suggesting that LAE1-induced clustering regulation may have a higher priority in secondary metabolic regulation than transcription factors. In another virulence factor bocinic acid (BOA) gene cluster, the FPKM of the entire BOA gene cluster including *BcPKS6* and *BcPKS9* was decreased. When the expression level of BOT and BOA clusters was downregulated, the virulence of *B. cinerea* was inevitably affected. This is consistent with the phenomenon in *B. cinerea* B05.10, where deletion of *lae1* directly impairs infectivity (Schumacher et al., 2015). In addition, this also reflects that although the key SM genes affected by BcLAE1 are located at different chromosomal locations, it tends to regulate the whole cluster and have no significant influence on the outside of the cluster region, while *BcabaR1* does not have the same expression pattern as *Bcaba1-4*, probably it is not in the ABA gene cluster or not connected to the cluster. This form of regulation is similar to the regulation of *Aspergillus nidulans* on the ST gene cluster; Chip experiment showed that the LaeA-mediated heterochromatin state reversal was limited to the cluster region, and the sequence outside the cluster remained heterochromatin under all metabolic conditions (Reyes-Dominguez et al., 2010). Therefore, we hypothesized that BcLAE1 might play the role of methyltransferase, because the way LAE1 regulates secondary metabolism by chromatin modification is conducive to the easy acquisition and coordinated regulation of gene clusters.

So far, no methylation substrate of LAE1 has been found. Patananan et al. (2013) used [3H] AdoMet in *Aspergillus nidulans* to observe the automethylation of LaeA only on methionine residues, but there was more and more evidence that LAE1 was involved in histone modification. In *Penicillium oxalicum*, PoTup1 recruited methyltransferase PoLaeA to modify the chromatin structure of the upstream region of the cellulose decomposition gene, thereby promoting the binding of transcription mechanisms to activate the corresponding cellulose gene expression (Zhang et al., 2022). In *Trichoderma reesei*, LAE1 induces gene expression by changing the H3K4me3 marks (Karimi-Aghcheh et al., 2013). Therefore, we mutated the SAM domain of BcLAE1, but the yield of ABA was not significantly increased in Δ*Bclae1*-C^SAM^ transformant compared with the Δ*Bclae1* transformant, suggesting that the SAM domain of BcLAE1 was very important to its function. In subsequent WB validation experiments, the deletion of *Bclae1* increased the level of H3K9me3 modification and decreased the level of H3K4me2 modification, confirming that BcLAE1 is involved in chromatin landscape regulation. Meantime, the histone modification marks did not change significantly in the *Bclae1*-OE-X4 transformant. The level of H3K4me2 was recovered in Δ*Bclae1*-C transformant, while the level of H3K9me3 modification did not change significantly.

At the same time, we observed differences in growth, morphology, and reproductive development of different *Bclae1* transformants. With the reduced expression of melanin key synthetic genes, the mycelium of the Δ*Bclae1* transformant appeared white compared with that of TB-31. Analysis of related pigment genes revealed increased transcriptional level of the putative UV-light-sensing cryptochrome *Bccry2* and decreased transcriptional level of the conidial positive regulator *Bcltf2*, which would result in reduced conidia, reduced melanin, and excess aerial hyphae. This coincides with the phenotypic characteristics of the Δ*Bclae1* transformant. However, RT-qPCR data showed that the expression level of *Bccry2* was not increased, indicating that Bccry2 was not the main factor causing this phenotype. Of course, studies have shown that the deletion of *Bcatf1* would also result in impaired conidial production, abnormal growth, and thick layers of aerial hyphae (Temme et al., 2012), but its expression level had not changed significantly. Meanwhile, under DD and LD photoperiods, the Δ*Bclae1* transformant lacked sclerotia formation in addition to the reduced number of conidia, so it was female sterile. The Δ*Bclae1*-C transformant not only had mycelial morphology and color close to TB-31, but also compensated for the functional defect of sclerotia formation and increased conidia production compared with the Δ*Bclae1* transformant. A recent study by Hou et al. (2020) found that H3K4me2/3 controlled the pathogenic development and virulence of *B. cinerea*, affecting spore germination, infection pad (IC) formation, appressorium formation, and stress adaptation. An interesting phenomenon is that in Δ*Bclae1* transformant, H3K4me2 modification is decreased, mycelium morphology is changed, and sporulation ability is significantly decreased. And in Δ*Bclae1*-C transformant, H3K4me2 modification was restored, the morphology of mycelium was close to TB-31, and the sporulation ability was recovered. However, the level of H3K9me3 modification was increased in the Δ*Bclae1* transformant but not significantly decreased in the Δ*Bclae1*-C transformant, and ABA production was also not fully restored.

We conjectured that ectopic complementation of BcLAE1 affected the regulation of histone modification and did not completely reverse the formation of heterochromatin, thus affecting the expression of SM clusters, which may be one of the reasons for the incomplete recovery of ABA production in Δ*Bclae1-C* transformant. As the ectopic expression of SM activators may lead to the separation of SM gene expression from the usual signaling pathway, such as the addition of an additional transcription regulator aflR outside the *A.nidulans* ST gene cluster restores ST production in Δ*laeA* transformant (Bok et al., 2006). Brahag et al. constructed multiple ectopically integrated inverters using the hypothesized activation gene *apdR*, successfully activating silenced clusters (aspyridinone) under unknown natural expression conditions (Bergmann et al., 2007). At the same time, another possibility cannot be ruled out, that the ectopic expression of *Bclae1* may affect its connection with its interaction partners, thereby interfering with the cooperative regulation of secondary metabolic networks, such as linking with BcVEL1 and BcVEL2 to form a heterotrimeric complex, or interacting with the homologous protein of the chromosome segregation protein Spc105 (Zhi et al., 2019), etc. Of course, studies have shown that ectopic *laeA* complementation in *Aspergillus fumigatus* restored NRPS gene expression levels (Perrin et al., 2007). Therefore, in view of the specificity of LAE1 protein, we will continue to explore how it regulates histone modification.

In conclusion, we found that BcLAE1 is essential for ABA biosynthesis in *B. cinerea* TB-31, and its deletion leads to the downregulation of the overall expression level of ABA synthesis gene cluster, while this aggregated regulation is widely distributed on chromosomes. In addition, BcLAE1 is involved in histone modification regulation, which affects the changes of H3K4me2 and H3K9me3 modification markers, which may also be one of the reasons affecting ABA and other secondary metabolisms synthesis. The regulatory pattern of LAE1 targeting secondary metabolic gene clusters found in this study is instructive for the subsequent mining of LAE1 targets. Our future work will compare the ABA yield of *Bclae1 in situ* complementation and ectopic complementation, and understand whether the difference of *Bclae1* insertion position has an effect on the location preference of cluster gene regulation from the point of view of chromatin modification.

## Data availability statement

The data presented in the study are deposited in the Science Data Bank (Science DB) repository, accession number 10.57760/sciencedb.01984. This data can be found here: http://doi.org/10.57760/sciencedb.01984.

## Author contributions

ZW designed and performed the experiments and prepared the original draft. ZW and DS designed the manuscript. ZW and D-bC analyzed the data. All authors contributed to the article and approved the submitted version.

## Funding

This research received financial support from the National Natural Science Foundation of China (32070059). We acknowledge funding from the Open Research Fund of Key Laboratory of Environmental and Applied Microbiology, Chengdu Institute of Biology, Chinese Academy of Sciences (KLEAMCAS201901).

## Conflict of interest

The authors declare that the research was conducted in the absence of any commercial or financial relationships that could be construed as a potential conflict of interest.

## Publisher’s note

All claims expressed in this article are solely those of the authors and do not necessarily represent those of their affiliated organizations, or those of the publisher, the editors and the reviewers. Any product that may be evaluated in this article, or claim that may be made by its manufacturer, is not guaranteed or endorsed by the publisher.
