# Supplementary material for: The BcLAE1 is involved in the regulation of ABA biosynthesis in Botrytis cinerea TB-31
Source: Front Microbiol. 2022 Aug 4;13:969499. doi: 10.3389/fmicb.2022.969499 (PMC9386520; doi:10.3389/fmicb.2022.969499)
Supplement: Supplementary file 1 [file Data_Sheet_1.zip › Supplementary_Material.docx]

Supplementary Material

**Figure S1.** HPLC compound profiles. HPLC detection chromatogram of ABA standard sample **(A)**, *Botrytis cinerea* TB-31 cultured for 12 days **(B)**, and Δ*Bclae1* transformant cultured for 12 days **(C)**.

**Figure S2.** Identification of the LAE1 ortholog in *Botrytis cinerea* TB-31 and validation of knock-out mutant. **(A)** Sequence diagram of *Bclae1* gene in the *Botrytis cinerea* TB-31 genome. The curves are the 6 introns that interrupt the ORF. Deletion of 4 bases "TACT" at the end of the 3'UTR compared to *Botrytis cinerea* B05.10. **(B)** Construction of *Bclae1* mutant. *Bclae1* gene conserved domain was replaced by hygromycin expression cassette (*PoliC*::*hph*). **(C)** Diagnostic PCR to validate knock-out results. The primer pairs used in lane 1 is Hph-F1/Hph-R1, the primer pairs used in lane 2 is *Lae1*-5-F1/Hph-YZ-R1, the primer pairs used in lane 3 is Hph-YZ-F1/*Lae1*-3-R1, the primer pairs used in lane 4 is *Lae1*YZ-F1/*Lae1*YZ-R1.

**Figure S3.** Validation of transcriptome expression patterns by RT-qPCR. These selected genes are *Bcbot1*, *Bcbot2*, *Bcbot3*, *Bcboa3*, *BcPKS13*, *Bccit3*, *Bcfas1*, *Bcfas2*, *BcACS*, *BcFBA*, *BcICL*, *BcMLS*, *BcNRPS5*, *BcPKS19*, *BcPYC*, BC1G_05489, BC1G_11623. The relative transcriptional levels of selected genes were obtained after normalization to the constitutive tubulin reference gene (BC1G_05600) at 6 days. The relative values for selected genes transcription at 6 days in TB-31 were arbitrarily assigned as 100%. Shown are means and SEM, n =3 independent biological replicates. Asterisks indicate significant differences in transcription levels between Δ*Bclae1* transformant and TB-31 (*, P < 0.05; **, P < 0.01; ***, P < 0.001).

**Figure S4.** *Bclae1* overexpression reduced ABA synthesis in *Botrytis cinerea* TB-31 transferred into pCBh1 empty vector. Twelve *Bclae1* overexpression mutants were randomly selected to grow on PDA. Samples for quantitative determination of ABA production were collected at day 7. The error bars indicate the SEM for three replicate cultures (n = 3). Asterisks indicate significant differences in ABA production between selected overexpression mutants and TB-31 (P < 0.05).
