# Supplementary figures and images for: The BcLAE1 is involved in the regulation of ABA biosynthesis in Botrytis cinerea TB-31
Source: Front Microbiol. 2022 Aug 4;13:969499. doi: 10.3389/fmicb.2022.969499 (PMC9386520; doi:10.3389/fmicb.2022.969499)

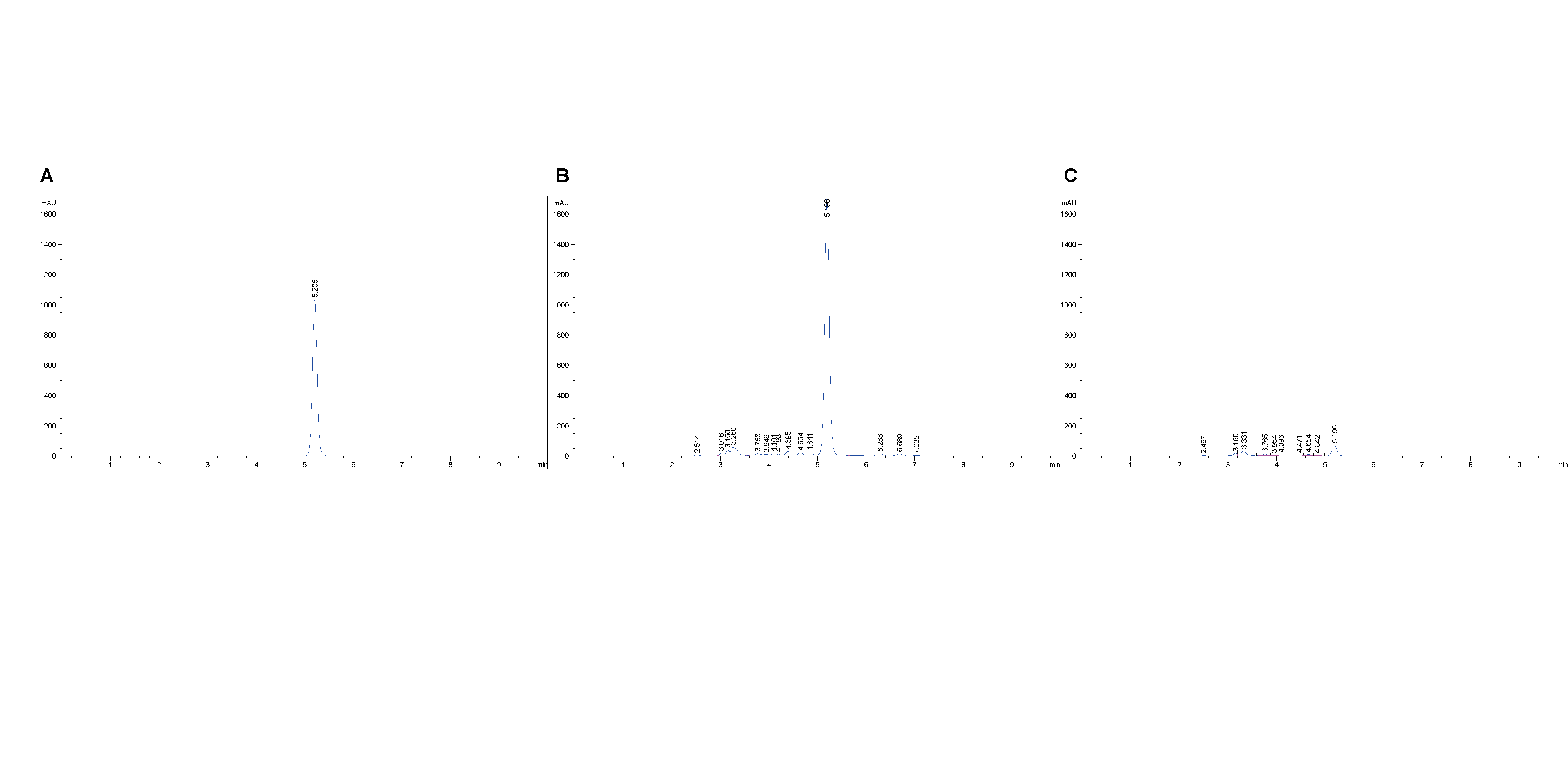

Supplement: Supplementary file 1 [file Data_Sheet_1.zip › Sfigure-S1.tif]

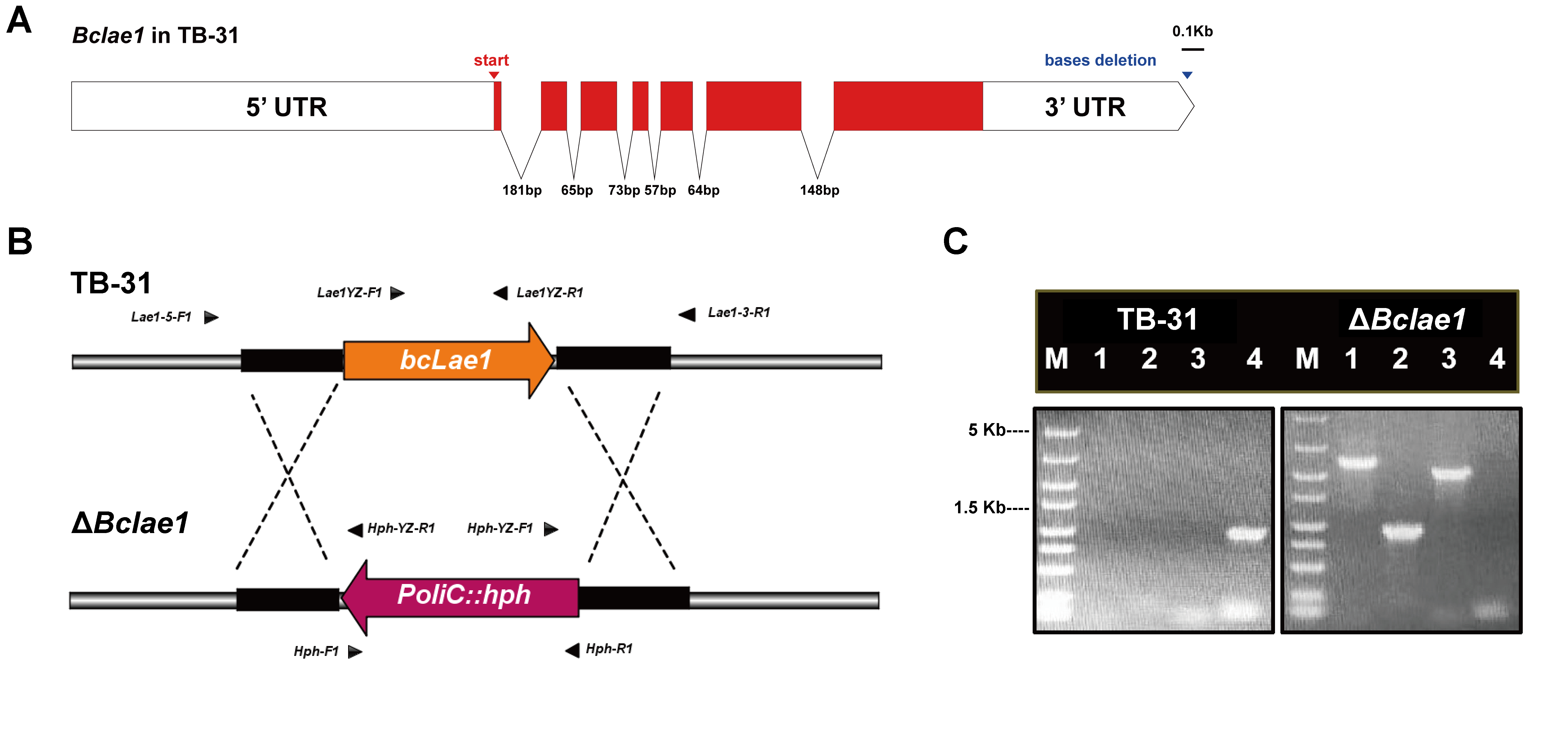

Supplement: Supplementary file 1 [file Data_Sheet_1.zip › Sfigure-S2.tif]

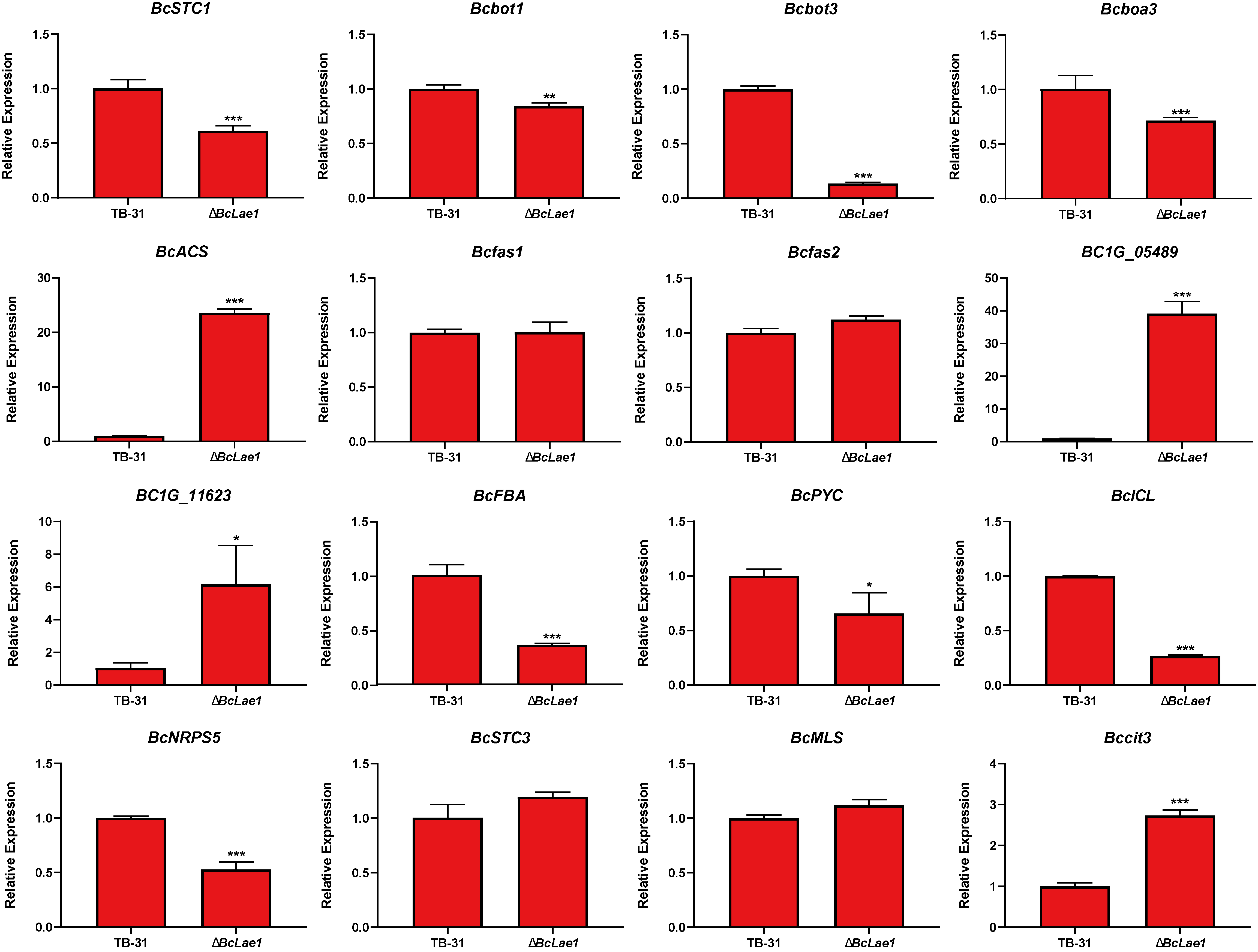

Supplement: Supplementary file 1 [file Data_Sheet_1.zip › Sfigure-S3.tif]

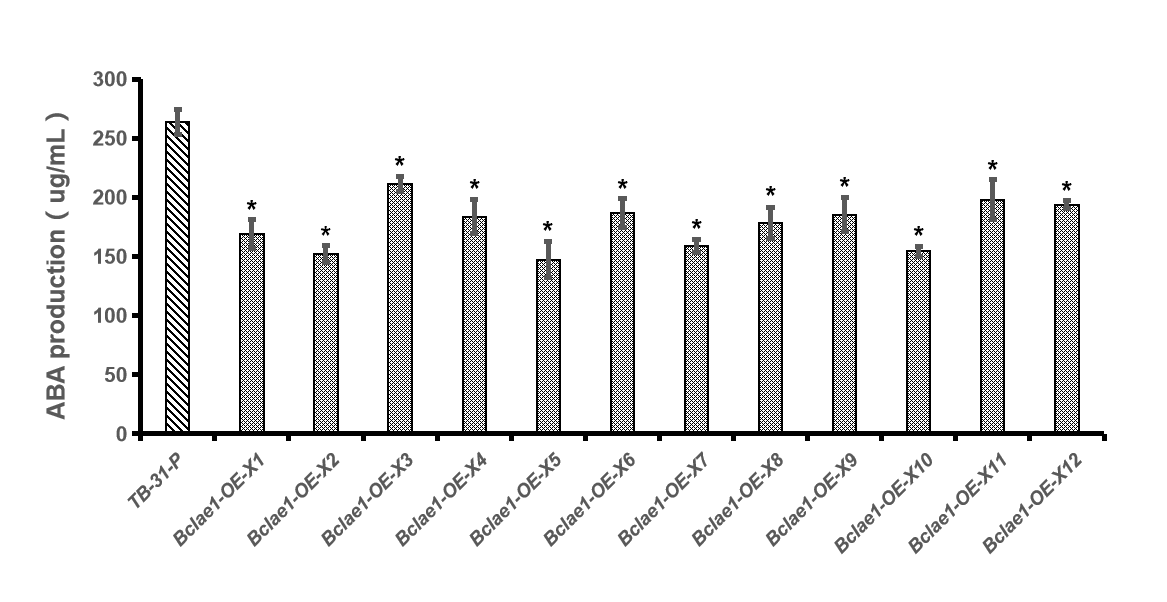

Supplement: Supplementary file 1 [file Data_Sheet_1.zip › Sfigure-S4.tif]
